# Obesity: assessment and treatment across the care continuum

**DOI:** 10.1080/07853890.2025.2521433

**Published:** 2025-06-26

**Authors:** Robert F. Kushner, Marla Shapiro

**Affiliations:** a Departments of Medicine and Medical Education, Northwestern University Feinberg School of Medicine, Chicago, IL, USA; b Department of Family and Community Medicine, University of Toronto, Toronto, Canada

**Keywords:** Obesity, chronic care, pharmacotherapy, behavioural intervention, patient-centred care, shared decision-making

## Abstract

**Background:**

Obesity is a chronic condition of dysregulated energy balance that is caused by a confluence of nutritional, neurological, hormonal and metabolic factors. Clinically, obesity is associated with myriad consequences to overall health. Treatment should be based on a shared decision-making process between the patient and their professional team that considers the stage of the disease, wellness goals and desired lifestyle and can include a combination of behavioural and lifestyle interventions, pharmacological therapies and surgery. The chronic nature of obesity necessitates adjustments in treatment plans to match the evolving needs and goals of the patient over time, establishing a ‘care continuum.’

**Methods:**

We conducted a broad, narrative literature search using PubMed for articles published on the assessment, diagnosis and treatment of adults with obesity.

**Results:**

In this narrative literature review, we outline evidence-based best practices for the diagnosis and assessment of obesity along with the various available treatment modalities. We also present considerations for treating patients with obesity with a focus on the selection of obesity medications based on severity, concurrent conditions, mechanisms of action and treatment goals. Finally, we discuss lifestyle management, the shift from weight loss to weight maintenance, and the implications of these changes in the care continuum.

**Conclusion:**

Continued, individualized treatment of patients with obesity from diagnosis to weight maintenance is imperative for sustained weight reductions, and strategies should be tailored to the changing needs of patients over time.

## KEY MESSAGES

- Obesity is a chronic condition that requires lifelong, multimodal management with lifestyle interventions, behavioural therapies and pharmacotherapy and/or surgery when indicated.
- Selection and initiation of treatments must be based on individual patient characteristics, comorbidities and goals.
- Changes in patient circumstances, health and goals over time must be reflected in a patient’s obesity management plan across their lifespan.

## Introduction

Obesity is a chronic and progressive disease of appetite dysregulation and energy imbalance characterized by the accumulation of adipose tissue that affects approximately 16% of adults globally [1]. Clinically, obesity is associated with a variety of complications and comorbidities, including type 2 diabetes, cardiovascular disease, obstructive sleep apnoea, metabolic dysfunction–associated steatohepatitis (MASH) and some forms of cancer [1,2].

The treatment of obesity relies on the principles of primordial, primary, secondary and tertiary prevention. Primordial prevention refers to population-based strategies to reduce the underlying contributors to obesity risk factors, such as the mass production and distribution of low-cost, energy-dense processed foods [3]. Primary prevention measures are aimed at disease occurrence and may include the promotion of improved psychological health and nutritional and physical activity–related behaviours associated with lowered risk of obesity [4]. Secondary prevention focuses on detecting and addressing diseases or health conditions at an early stage; examples include screening for obesity, evaluating for obesity-related complications and treatments with lifestyle modifications, psychological interventions, obesity medications and bariatric surgery in patients with a BMI ≥40 kg/m^2^, ≥35 kg/m^2^ with a comorbidity, or ≥30 kg/m^2^ with type 2 diabetes [2,4]. Finally, tertiary prevention includes treatments that limit the complications of obesity and their consequences on health [4].

Because obesity requires lifelong treatment, patients’ goals may change over time; therefore, lifestyle and medical approaches to achieve and maintain those goals need to continuously shift over the course of the patient’s life [5]. It is essential for health care professionals to be familiar with the principles of obesity prevention and treatment. This narrative literature review outlines evidence-based factors primary care professionals should consider when treating the evolving needs of patients with obesity over time, or establishing a ‘care continuum,’ especially when choosing obesity medications.

## Methods

We conducted a broad, narrative literature search using PubMed for articles published on the assessment, diagnosis and treatment of adults with obesity. Articles published between 2014 and 2024 were primarily included to ensure that this review includes updated and relevant recommendations to health care professionals. Articles written in a language other than English and conference abstracts were excluded.

## Discussion

### Neurophysiology of obesity

Obesity is a complex disease of energy imbalance involving dysregulated neurological, hormonal and metabolic factors [6]. Energy balance is regulated by the complex interaction between the homeostatic and hedonic brain pathways that control energy intake and expenditure [6]. The hypothalamus promotes energy balance by integrating peripheral hunger and satiety hormonal signals from the stomach, pancreas and intestines (called the ‘gut-brain axis’) and from adipose tissue [7]. For example, activation of pro-opiomelanocortin (POMC)-secreting neurons in the hypothalamus that is triggered by anorexigenic gut hormones and adipose-derived leptin promotes satiety [8]. Other mechanisms are implicated in obesity and energy balance, such as neurons in the hindbrain that inhibit food intake and the dopaminergic mesolimbic pathway that promotes food consumption for its rewarding properties regardless of metabolic state [9,10]. Dysregulation of the hypothalamic and dopaminergic systems, such as a greater hypothalamic volume and reduced dopaminergic signalling, have been observed in people with obesity [11,12]. Therefore, health care professionals should consider these underlying physiological factors in the diagnosis and management of obesity by recognizing how available treatment options interact with the neuroendocrine dysregulation observed in obesity.

### Assessment, diagnosis and treatment initiation for obesity

#### Assessment and diagnosis

Collecting a patient history, performing a physical examination, and obtaining laboratory testing are part of a comprehensive evaluation for the diagnosis of obesity [2]. A patient history should include information on the patient’s pattern of weight gain over time and past weight reduction attempts, physical and mental health, medications, surgery, life circumstances and behaviours, and barriers to weight loss [2,13]. Laboratory testing should be individualized to the patient and assess measures of glycaemia (e.g. fasting glucose and haemoglobin A1C), lipid levels, thyroid-stimulating hormone and liver enzymes, among others. Other diagnostic tests may include sleep studies for patients with suspected sleep apnoea and liver imaging for patients with type 2 diabetes or at risk for metabolic dysfunction–associated liver disease as appropriate based on patient history and blood test results for elevated fibrosis 4 (FIB-4) index score [2].

Body mass index (BMI) is the most common screening metric used to identify obesity and is strongly associated with population-based risk of cardiometabolic diseases [14]. Cut points for the diagnosis of overweight and obesity are racially dependent. For example, a BMI of 25 kg/m^2^ to 29.9 kg/m^2^ is classified as overweight and BMI ≥30 kg/m^2^ is classified as obesity for White people [15,16]. For people of Asian descent, a BMI of 23 kg/m^2^ to 24.9 kg/m^2^ is classified as overweight and BMI ≥25 kg/m^2^ is classified as obesity [15]. However, because BMI does not directly measure body composition or consider body fat distribution, patients with low proportions of body fat and high muscle mass may have a BMI in the overweight or obesity range [17]. Therefore, including another anthropometric measurement such as waist circumference or waist-to-height ratio provides additional information regarding the location of adiposity and risk for metabolic disease in patients with a BMI <35 kg/m^2^ [2].

Waist circumference is strongly associated with visceral fat and increased morbidity and mortality and identifies patients with a high-risk phenotype [18]. For White women, waist circumference thresholds are ≥90 cm for overweight and ≥105 cm for obesity, whereas waist circumference thresholds for men are ≥100 cm for overweight and ≥110 cm for obesity [18]. For people of Asian descent, waist circumference thresholds for obesity are lower, defined as ≥90 cm for men and ≥80 cm for women [2]. Alternatively, a waist-to-height ratio of ≥0.5 may also accurately predict cardiometabolic risk and does not depend on age-, sex-, or race-specific thresholds [2].

Although measuring body fat is not a standard of care, it may be useful in subpopulations where BMI misclassifies patients, such as young muscular individuals or elderly patients with sarcopenia [2,19]. The most commonly used clinical instruments for estimating body fat and lean body mass are bioelectrical impedance analysis (BIA) and air displacement plethysmography [2]. Percent body fat thresholds range from 30% to 34% for women and 25% to 29% for men with overweight and ≥35% for women and ≥30% for men with obesity [2]. Issues such as availability, expense, office space and inaccuracy (e.g. inattention to standard measurement conditions for BIA) are obstacles to the use of these instruments in the outpatient setting [2]. Recent technological advancements, such as computer algorithms that use smartphone scanning to estimate a person’s percent body fat, called digital anthropometry, could eventually increase the feasibility of body fat percentage as an evaluative tool [20].

#### Treatment initiation

As a chronic disease, obesity requires long-term treatment [2]. Thus, health care professionals should be prepared to treat patients along the entirety of a care continuum, from assessment and diagnosis to treatment and long-term maintenance. A patient-centred approach to obesity treatment focuses on shared decision-making between the health care professional and patient and cultivates a supportive, nonjudgmental environment [21]. This shared approach may include asking permission from a patient to discuss their weight and offering a private location to measure their weight [21]. Health care professionals should discuss expectations and goals for weight loss with their patients with obesity using motivational interviewing techniques to establish a collaborative, goal-directed approach to behavioural modification [2]. Because patients with obesity often underestimate the impact of excess weight on their health, these discussions should also reflect the benefits of weight loss as a therapeutic strategy to decrease obesity-related health complications [22,23]. For example, a 5% to 10% body weight reduction is associated with reduced risk factors for obesity-related complications, such as lower blood pressure, blood glucose and blood triglycerides [24,25]. Similarly, for patients with a BMI ≥40 kg/m^2^, a BMI ≥35 kg/m^2^ with a comorbidity, or type 2 diabetes, a referral for bariatric surgery may be appropriate to achieve higher weight-loss goals [2,4].

Best practice for obesity treatment involves a multifactorial approach based on 4 pillars: nutrition therapy, physical activity, behavioural modification and pharmacotherapy [4,26,27]. In a primary care setting, this is commonly directed by a primary care professional but can also involve coordination with a health care team, including a registered dietitian, exercise physiologist, a diabetologist and a behavioural health consultant [28–31].

Multifactorial treatment can include the use of the ‘5 As’ framework of obesity management as recommended by the Obesity Medicine Association and Obesity Canada: (1) ask permission to discuss body weight and explore readiness for change; (2) assess BMI and waist circumference and explore the drivers of excess weight; (3) advise the patient about obesity health risks, the benefits of weight reduction, treatment options and long-term strategies; (4) agree on realistic goals, behavioural changes and treatment plan; and (5) assist in identifying barriers, finding appropriate health care professionals and accessing resources and arranging regular follow-up appointments [2,32]. Behavioural modifications can include self-monitoring of weight, diet and physical activity, nutritional counselling (e.g. reducing calorie intake, increasing fruits and vegetables), physical activity recommendations (e.g. accumulate 30 min of daily brisk walking), identifying barriers to weight loss, peer support, and relapse prevention [33]. Interventions can be individual, group-based, technology-based, or a combination of these; most programs include at least 12 individual sessions in the first year, while group programs often offer almost twice that [33].

When developing a treatment plan, primary care professionals should consider barriers associated with the social determinants of health, which may impact the resources available to patients [34]. For example, a cross-sectional study of data from the Behavioural Risk Factor Surveillance System telephone survey from 2003 to 2013 examined the association of county-level factors with the BMI of people living in those communities [35]. This study demonstrated that people who live in low-income communities with limited access to food and physical activity have higher BMIs than people who live in high-income communities with greater access to active commuting, which increases physical activity [35]. Moreover, some communities lack safe spaces for residents to engage in physical activity, which may contribute higher levels of obesity [34]. Decreased access to healthful nutrition and safe places for physical activity, as well as limited access to obesity treatment as a result of economic, educational, cultural and environmental factors may therefore limit treatment options available to some patients [34]. Primary care professionals should therefore be prepared to discuss such limitations with patients and provide recommendations to community programs, such as those that help provide healthful foods to patients with limited access or safe places to engage in physical activity [34].

Patients who are not meeting their health-related goals through lifestyle modification approaches alone may benefit from the addition of an obesity medication to enhance weight loss [26,36]. If the medication results in a weight reduction of ≥5% without safety or tolerability concerns, the continuation of that medication is recommended since a lower initial weight loss is associated with a less successful outcome [26,36]. Alternative treatment approaches, such as a different obesity medication or bariatric surgery, should be considered if patient goals are not met [26].

### Pharmacotherapies

#### Available pharmacotherapies and their effects

The American Association of Clinical Endocrinologists, Obesity Canada and the Endocrine Society recommend pharmacotherapy for patients with a BMI ≥30 kg/m^2^ or ≥27 kg/m^2^ with obesity-related complications, such as type 2 diabetes, cardiovascular disease, or metabolic syndrome [4,26]. Of note, the US Food and Drug Administration has recently removed a BMI cut point for the prescription of 3 obesity medications, including phentermine-topiramate ER, semaglutide and tirzepatide [37–39]. Approved obesity medications work through a variety of mechanisms of action and brain systems, including (but not limited to) the hypothalamic system, mesolimbic system and hindbrain. Nutrient-stimulated hormone-based pharmacotherapies, such as liraglutide, semaglutide and tirzepatide, target the glucagon-like peptide-1 receptor (GLP-1R), with tirzepatide also targeting the glucose-dependent insulinotropic polypeptide (GIP). GLP-1R agonists are believed to promote POMC activity in the hypothalamus to increase satiety, though some studies have suggested that hypothalamic GLP-1R activation is not necessary to induce weight-loss effects [38,40–45]. Hindbrain and mesolimbic areas are also implicated in GLP-1R agonist–mediated effects on food intake and energy balance [46–48]. Sympathomimetic medications such as phentermine + topiramate extended release (ER) increase the activity of norepinephrine and dopamine in the hypothalamus and other brain regions to stimulate POMC activity [49]. In the fixed-dose, ER combination of naltrexone and bupropion (NB-ER), bupropion acts as a norepinephrine and dopamine reuptake inhibitor to stimulate POMC activity and naltrexone acts as an opioid antagonist to prevent autoinhibition of POMC cells to promote satiety [50].

Patients’ response to obesity medications is heterogenous. Generally, a patient is considered a ‘responder’ to an obesity medication if they lose ≥5% of their body weight within 3 months of starting a maintenance dose [26]. For example, responders lost approximately 12% of their body weight at 56 weeks with phentermine-topiramate ER combined with lifestyle modification counselling compared with 1% to 2% reductions in body weight with placebo in a randomized, controlled trial [51]. Similarly, according to a post hoc analysis of the 4 randomized, controlled NB-ER trials, responders to NB-ER treatment lost approximately 9% to 14% of their body weight at 56 weeks vs 1% to 2% with placebo [52]. This range in response may in part be due to methodological differences. For example, patients in the COR-I, COR-II and COR-DM trials received information on nutrition and physical activity, in addition to NB-ER or placebo, but patients in the COR-BMOD trial participated in an intensive program of diet, physical activity and behaviour modification therapy in addition to NB-ER or placebo and had a greater weight reduction than patients in the trials without intensive behaviour therapy [36,52–55]. Treatment with tirzepatide along with lifestyle modification was associated with weight reductions of ≥20% in 57% of participants over 72 weeks compared to 2% body weight reductions with placebo treatment in the randomized, controlled SURMOUNT 1 clinical trial [56]. Table 1 shows the effects of these FDA-approved obesity medications, as well as their effects on long-term weight maintenance. Importantly, these outcomes are based on evidence from clinical trials, which each included different lifestyle modification plans and may have limited generalizability to the patients being treated in a primary care setting.

**Table 1.** Weight loss and maintenance with pharmacotherapy for obesity.

Obesity medication	Mean percent body weight reduction in clinical trials	Mean long-term weight percent body weight reduction in clinical trials
Phentermine-topiramate ER	9%–12% in 56 weeks [51,65]	9%–11% in 108 weeks [65]
NB-ER	5%–14% in 56 weeks [52]	NA
Liraglutide	8% in 56 weeks [96]	6%^a^ in 68 weeks [97]
Semaglutide	15%–16% in 68 weeks [58,95]	15% in 104 weeks [98]
Tirzepatide	17%–23% in 72 weeks [56]	25.8% in 88 weeks [57]

^a^ Body weight reduction is in addition to 6% weight loss prior to randomization.

ER, extended release; NA, not available; NB-ER, fixed-dose, ER combination of naltrexone and bupropion.

Weight-loss outcomes with obesity medications must be balanced against safety and tolerability considerations. Obesity medications are associated with a range of adverse events that vary by medication and may include nausea, diarrhoea, constipation, vomiting, dizziness and dry mouth among others [53,57,58]. Many such adverse events are generally considered mild to moderate in severity, occur during the dose escalation period and can generally be managed by avoiding high-fat foods and caffeine, eating smaller meals, exercising regularly, incorporating fibre-rich foods, increasing water intake and remaining upright after meals [59–63]. Furthermore, slowing or prolonging the dose escalation is recommended to improve the tolerability of obesity management medications [26].

### Pharmacotherapy selection

The selection of obesity medications should be personalized and consider individual patient characteristics and circumstances. Patient history of previous weight-loss attempts, previous treatments and their effectiveness and treatment goals are relevant to the selection process [2,4]. Furthermore, the principles of chronic disease management hold that patient goals and values should be incorporated into obesity prevention and treatment decisions; thus, the timing, intensity and types of treatments initiated will vary depending on multiple factors [4].

For patients with a goal of 5% to 10% body weight reduction, treatment should focus on lifestyle modifications and obesity medication to improve risk factors and prevent disease progression [64]. For these patients without severe obesity-related health consequences, medications that produce 5% to 15% reductions in body weight, such as phentermine-topiramate ER and NB-ER, may be appropriate [52,53,65–68]. A higher weight-loss goal of ≥15% may be appropriate for patients with conditions such as MASH, severe obstructive sleep apnoea, or existing cardiovascular disease [64]. For these patients, obesity medication such as weekly injectable semaglutide or tirzepatide that produce 17% to 23% reductions in body weight may be more appropriate [56,69]. Thus, it is imperative to personalize treatment to each patient’s situation and needs. While pharmacotherapy should be used in conjunction with lifestyle modifications such as a healthful eating pattern and increased physical activity, some patients may benefit from more intensive behavioural programs in conjunction with pharmacotherapy. The use of intensive behaviour modification programs that may include individual and group sessions with registered dietitians, psychologists and exercise specialists in addition to pharmacotherapy can also have beneficial effects on weight loss and cardiometabolic improvements, as demonstrated in randomized, controlled clinical trials for NB-ER, liraglutide and semaglutide [36,70,71].

The shared decision-making process should also consider the treatment routes of available pharmacotherapies. While NB-ER and phentermine-topiramate ER are oral medications, liraglutide, semaglutide and tirzepatide are currently only available for obesity treatment in injectable formulations [2]. For some patients, an oral medication may be preferrable to an injectable one.

Some medications may be more appropriate for patients with specific obesity phenotypes. In a single-centre, pragmatic obesity clinical trial, participants with obesity were classified into four obesity phenotypes based on validated clinical tests and questionnaires and included eating behaviour, affect and physical activity, among other characteristics [72]. NB-ER was used for patients with obesity who exhibited emotional eating (high depression and anxiety scores), phentermine-topiramate ER for patients with abnormal satiation (high calories required to reach fullness), liraglutide for patients with abnormal satiety (low duration of fullness) and phentermine for patients with low resting energy expenditure [72]. The phenotype-guided approach to pharmacotherapy was associated with a 16% body weight reduction compared with 9% using non–phenotype-guided treatment (i.e. usual care) [72]. Efforts are underway to identify reliable predictors of response to obesity treatments [73].

Certain medications may also be preferable in patients depending upon comorbid conditions. Patients with obesity and co-occurring type 2 diabetes may benefit from an antidiabetic medication known to promote weight loss, such as a GLP-1R agonist or GIP/GLP-1R dual agonist [21,26]. Conversely, sympathomimetic medications such as phentermine-topiramate ER are not recommended for patients with cardiovascular disease, glaucoma, or patients taking monoamine oxidase inhibitors [2,26]. Some guidelines recommend the use of NB-ER in weight-loss interventions for patients with obesity who are attempting to quit smoking and in patients with depression [74,75]. NB-ER contains bupropion, an approved treatment for depression and smoking cessation [50]. A small, open-label study found that NB-ER and behavioural treatment combined was associated with reduced nicotine withdrawal symptoms and a prevention of post–smoking cessation weight gain in people who smoked [76]. In a large, post hoc analysis of a multicentre, randomized, controlled trial, patients with overweight and obesity who take antidepressants had an increased likelihood of achieving 5% or 10% weight loss at 16 weeks and at 52 weeks with NB-ER vs placebo, with no differences in safety and tolerability compared with patients who do not take antidepressants [77]. For patients with obesity and migraine headaches, phentermine-topiramate ER may be considered due to the inclusion of the migraine medication topiramate [21]. Finally, the use of injectable GLP-1R agonist or GIP/GLP-1R–based medications is contraindicated in patients with a personal or family history of medullary thyroid carcinoma or patients with type 2 multiple endocrine neoplasia syndrome [2].

Another important consideration during shared decision-making is the cost associated with long-term obesity management across the care continuum, which can be a barrier to treatment for some patients due to economic and social factors. The financial cost of injectable medications such as tirzepatide ($550 to $1118/month), semaglutide ($717 to $1225/month) and liraglutide ($761 to $1281/month) can significantly burden patients [78,79]. Oral medications, such as NB-ER ($99 to $200/month) or phentermine-topiramate ER ($100 to $250/month) may be more affordable and therefore more appropriate for patients who lack insurance coverage for injectable medications [79–82].

### The continuum of care in chronic obesity treatment

#### Weight loss with pharmacotherapy and lifestyle modifications

After beginning obesity medications, patients may notice a reduction in food cravings, improvements in their control of eating and decreased binge eating [76,83–85]. During active obesity treatment, patients should be closely monitored for the rate of weight loss and occurrence of any adverse symptoms or signs. Typically, patients should aim for a ≥ 5% body weight reduction after 3 months of treatment at an effective dose [26]. Excessive muscle mass loss can lead to reduced resting metabolic rate, fatigue and weakness [86,87]. Therefore, measures to preserve muscle mass during weight loss should be considered, including aerobic and resistance training, along with increased dietary protein [4,88–92]. A patient’s rate of weight loss may determine the type of tissue mass reduction; a small study using segmental multifrequency BIA found that gradual diet-induced weight reduction alone promoted a preferential reduction in fat mass, while rapid weight loss was associated with a preferential reduction in muscle mass [93]. Similarly, a slower rate of weight loss may also help preserve bone density [94]. Thus, if patients experience rapid and excessive weight loss with an obesity medication, it is imperative to counsel them on healthy lifestyle behaviours and consider reducing the dose of the medication [90–92]. The efficacy and safety of the treatment plan should be assessed at least monthly for the first 3 months; more frequent ­contact (≥12 visits per year) is associated with improved outcomes, possibly *via* increased accountability [21,26].

#### Long-term weight maintenance

As with other chronic conditions, combined behavioural and pharmacological obesity treatment should continue after the achievement of the initial weight-related goals [4,26,74]. Obesity medications do not permanently alter the underlying neuroendocrine disruption associated with obesity, meaning that medications and lifestyle modifications should be continued long-term to promote weight maintenance and to prevent and treat obesity-associated health complications [4,26]. For example, in the SURMOUNT 4 randomized withdrawal trial, switching to placebo at 36 weeks led to an increased mean body weight and a reversal of initial cardiometabolic risk factor improvements, while continued treatment with tirzepatide led to increased weight loss and body weight maintenance through 88 weeks [57]. Weight regain has also been observed with the discontinuation of semaglutide in the STEP 4 randomized clinical trial in patients who switched to placebo at 20 weeks, while patients who continued semaglutide maintained and further decreased their body weight through 68 weeks [95]. At this time, there is no evidence-based guideline regarding long-term pharmacological management of obesity, and different obesity medications may be considered for weight maintenance. For example, a patient who achieved 15% to 20% body weight loss with tirzepatide or semaglutide may continue their current medication if it is tolerable and effective. If a patient is not able to continue their current medication for reasons such as cost, access or tolerability, an alternative medication such as oral NB-ER or phentermine-topiramate ER may be preferred to the discontinuation of all obesity medications to help the patient maintain weight long-term. Patients taking obesity management medications should continue to be seen regularly by their primary care professionals during maintenance treatment [26].

### Limitations and future directions

The present narrative review is limited in its scope and focuses primarily on clinical trial data, which may have limited generalizability to a real-world setting. Additional research and critical review of real-world experience studies may provide valuable information to guide primary care professionals in their discussions with patients regarding their treatment options. More research is also needed to examine the role of lifestyle interventions such as protein intake (e.g. the quality and quantity of protein needed) and physical activity (e.g. the type and intensity of exercise) in preserving muscle mass in high-risk patients. Moreover, additional studies are needed to determine which patients will respond best to a given obesity medication. Future studies should also examine a variety of approaches to maintain weight reduction long term to provide patients with options that fit their unique and changing situation across the continuum of care. Furthermore, future research should identify key treatment thresholds at which patients with obesity achieve cardiometabolic improvements. Identifying these thresholds will allow health care professionals to provide patients with targeted and personalized treatment recommendations to improve their overall health.

## Conclusions

Continuous treatment of patients with obesity across the care continuum, from diagnosis to weight maintenance, is crucial to achieving sustained weight loss. The weight-loss strategies developed for patients with obesity require careful consideration of patients’ characteristics, complications, comorbidities and wellness goals. Weight maintenance strategies are lifelong and may differ from active weight-loss strategies. Thus, patients and health care professionals should consider which treatments are most suitable to achieving sustainable weight maintenance across the entirety of the care continuum.

## Data Availability

Data sharing is not applicable to this article as no new data were created or analyzed in this study.
